# Expression of IL-10 and TGF-β1 in horses experimentally infected with *T. equi* merozoites is associated with antibody production but not modulation of pro-inflammatory responses

**DOI:** 10.3389/fimmu.2024.1370255

**Published:** 2024-05-13

**Authors:** Cynthia K. Onzere, Reginaldo G. Bastos, Richard P. Bishop, Carlos E. Suarez, Lindsay M. Fry

**Affiliations:** ^1^ Department of Veterinary Microbiology & Pathology, Washington State University, Pullman, WA, United States; ^2^ Animal Disease Research Unit, Agricultural Research Service, US Department of Agriculture, Pullman, WA, United States

**Keywords:** *Theileria equi*, merozoites, host-parasite relationship, IL-10, TGF-β1, antibodies

## Abstract

*Theileria equi* (*T. equi*) is an apicomplexan parasite that causes severe hemolytic anemia in equids. Presently, there is inadequate knowledge of the immune responses induced by *T. equi* in equid hosts impeding understanding of the host parasite relationship and development of potent vaccines for control of *T. equi* infections. The objective of this study was to evaluate the host-parasite dynamics between *T. equi* merozoites and infected horses by assessing cytokine expression during primary and secondary parasite exposure, and to determine whether the pattern of expression correlated with clinical indicators of disease. Our findings showed that the expression of pro-inflammatory cytokines was very low and inconsistent during both primary and secondary infection. There was also no correlation between the symptoms observed during primary infection and expression of the cytokines. This suggests that the symptoms might have occurred primarily due to hemolysis and likely not the undesirable effects of pro-inflammatory responses. However, IL-10 and TGF-β1 were highly expressed in both phases of infection, and their expression was linked to antibody production but not moderation of pro-inflammatory cytokine responses.

## 1 Introduction

*Theileria equi* (*T. equi*) is an obligate intracellular hemoprotozoan parasite and a major causative agent of equine piroplasmosis (1). *T. equi* infection is characterized by severe hemolytic anemia that causes acute morbidity and death in some cases (2–4). The parasite has a wide global distribution (5) and is associated with extensive economic losses (6) that is exacerbated by the lack of a vaccine and effective therapeutics especially in cases of co-infection with *Theileria haneyi* (7).

*T. equi* has a dixenous life cycle which includes a sexual stage in the tick vector and asexual stages in the equid host (8). Parasite transmission involves the transfer of sporozoites from infected ticks to the equid hosts through saliva during tick feeding, The incoming *T. equi* sporozoites are able to recognize and invade peripheral blood mononuclear cells (PBMCs) of the equid host (8, 9), where they undergo schizogony prior to differentiation into merozoites (8). Cell lysis then occurs, leading to the release of merozoites that subsequently invade erythrocytes, where the parasites reproduce asexually (8). Clinical manifestation of *T. equi* infection is associated with the erythrocytic developmental stages in equid species involving massive erythrocyte destruction by the parasite, leading to hemolytic anemia. There is scanty knowledge on the host-parasite dynamics that occur in the pre-erythrocytic phase (9). Additionally, very little is also known about host immune responses to *T. equi* infection and bridging this knowledge gap is key in understanding the relationship between the parasite and its hosts. Although humoral immune responses have previously been demonstrated during *T. equi* infection (2, 10–13), there is a scarcity of information regarding the cell mediated immune responses that are induced in equids in response to infection. Cytokines are critical regulators of immune responses, and their quantification provides insights into cell mediated immune responses (14–17). For instance, studies have shown that pro-inflammatory cytokines (15) are produced during the blood infective stages of Plasmodium infection in order to control the parasite in the mammalian host. Production of anti-inflammatory cytokines (15) regulates these responses to mitigate severe morbidity and mortality (18–21). Similar observations have been made during natural infection of *Theileria annulata* (*T. annulata*) where an increase in anti-inflammatory cytokine levels are observed (22). Studies on *Theileria parva* (*T. parva*) have also shown a direct relationship between parasite DNA load, severity of disease and pro-inflammatory cytokines mRNA (23). Despite the fact that strong inflammatory responses are observed during *T. parva* infection, the production of anti-inflammatory cytokines and especially IL-10 have also been described as a potentially protective measure against lethal infection (23–26). Similarly, a study conducted on horses that were naturally infected with *T. equi* showed significant production of both pro- and anti-inflammatory cytokines in a parasite load dependent manner (27). However, and to our knowledge, no study has been conducted so far to assess the cell mediated immune responses that occur in response to the merozoite stages of *T. equi* infection. The objective of this study was to evaluate disease dynamics in experimentally infected horses during primary and secondary *T. equi* infection and to determine whether there is any correlation between the observed infection dynamics and the cytokines produced.

## 2 Materials and methods

### 2.1 Statement of ethical approval

All procedures were performed in accordance with the protocols approved by the Institutional Animal Care and Use Protocol Committee of the University of Idaho (protocol #2018-19). All procedures adhered to the U.S. National Institutes of Health (NIH) Guide for the Care and Use of Laboratory Animals.

### 2.2 Experimental animals

Six Welsh cross horses (HO-397, HO- 403, HO-394, HO-402, HO-396 and HO-398) were used in this study. All the horses were more than 6 months of age.

### 2.3 Parasite

*T. equi* Florida strain was used in this study. This is the reference parasite strain and its genome sequence has been published in GenBank (GenBank ID number: 11168).

#### 2.3.1 Infection of horses with T. equi blood stabilate and sample collection

**Figure 1:**
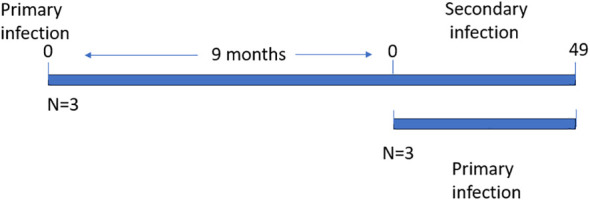
Schematic showing the timelines of primary and secondary *T. equi* infection.

The horses were divided into two groups of three animals each i.e., primary (acutely), and secondary (persistently) infected groups. All the animals were intravenously infected with two ml of the *T. equi* Florida strain erythrocyte stabilate that is constituted of merozoites at 16% parasitemia as previously described (28). The secondary infected animals (HO-397, HO-403 and HO-394) had previously been exposed to the parasite nine months before re-infection and the primary infected animals (HO-402, HO-396 and HO-398) had no prior exposure to the parasite (**Figure 1**). Two ml of blood samples were collected daily in the 5 ml BD Vacutainer^®^ tubes (The Becton, Dickinson and Company, Franklin Lakes, NJ) with EDTA as an additive for DNA extraction and subsequent evaluation of parasite DNA load, and hematocrit measurement. The samples were collected daily up to 30 days post-infection (DPI) after which samples were collected every other day. This is because peak parasitemia, severe hemolysis and pyrexia occur within this period during acute infection. This allowed us to monitor the animals and initiate treatment in a timely manner to manage disease related symptoms once they arose.

Ten ml of blood were collected in BD Vacutainer® tubes without anti-coagulant to obtain sera that were used for evaluation of cytokine expression. These samples were collected at 7-days intervals between 0 and 49 DPI as previously described (12, 13) with the exception of 35 DPI. Day 0 comprised of samples collected from the acutely infected animals prior to infection and from the persistently infected animals prior to the primary infection. The samples were collected at 7-days intervals because antibody responses and thus immune responses have previously been detected as early as 7 DPI with peak levels observed at approximately 30 DPI (29) depending on the quantity of infecting parasites.

#### 2.3.2 Assessment of parasite DNA load, hematocrit and febrility during T. equi infection

Disease progression was assessed by evaluation of parasite DNA load, hematocrit, febrility and physical symptoms such as anorexia, lethargy, malaise, pale mucous membrane, diarrhea, and tachycardia. Assessment of parasite DNA load on these samples was previously described (13). Briefly, DNA was extracted from blood collected in the BD Vacutainer® EDTA tubes, at 7-days intervals using the Qiagen DNeasy Blood & Tissue Kit (Qiagen, Hilden, Germany) according to the manufacturer’s instructions. *T. equi* quantitative PCR (qPCR) was then performed as previously described (30) and the data was reported as *T. equi* ema-1 copy number (#)/µg DNA (13).

Rectal temperature and hematocrit/packed cell volume (PCV) were analyzed daily for 30 DPI after which samples were collected every other day unless fever and/or physical symptoms were observed. Assessment of hematocrit was performed using the ProCyte DX™ (IDEXX Laboratories, Inc., Westbrook, ME) in accordance with the manufacturer’s instructions, and the output was visualized using the IDEXX VetConnect PLUS software.

#### 2.3.3 Evaluation of cytokine expression during the merozoite stages of T. equi infection

A multiplex assay was used to assess the presence of cytokines in the serum samples collected from both groups of horses. The EMD Millipore’s MILLIPLEX® MAP Equine Cytokine Magnetic Bead Panel kit (Millipore Sigma, Burlington, MA) was customized to evaluate the simultaneous expression of pro-inflammatory cytokines (IFN-γ, IL-1β, IL-6, IL-12 (p70), IL-17A and TNFα), anti-inflammatory cytokines (IL-10 and IL-4) and a chemokine (RANTES). The analysis was performed using neat serum samples, in duplicates, and in accordance with the manufacturer’s instructions. The Luminex® 200™ System (Luminex, Austin, TX) was used to measure the analytes, and the xPONENT® software (Luminex, Austin, TX) was used for data acquisition and calculation of the mean concentration of cytokines using the four-parameter logistic (4PL) analysis. The Belysa® Immunoassay Curve Fitting Software (Millipore Sigma, Burlington, MA) was used to further visualize the standard curve and the distribution of the samples, standards, and controls.

### 2.4 Evaluation of TGF-β1 expression

Since equine TGF-β1 is not available as an analyte in Millipore’s MILLIPLEX® MAP Equine Cytokine Magnetic Bead Panel kit, the horse transforming growth factor beta1(TGFB1) ELISA Kit (Cusabio, Houston, Tx) was used to assess expression of the cytokine in the serum samples. The assay was performed in accordance with the manufacturer’s instructions and like the Milliplex assay, 4PL was used to determine TGF-β1 concentrations in the samples and the Assayfit pro software (AssayCloud, Nijmegen, Netherlands) was used to perform the analysis.

### 2.5 Assessment of equi merozoite antigen-1 antibody production during primary and secondary T. equi infection

The *Theileria equi* antibody test kit (VMRD, Pullman) was used to perform competitive ELISA for the detection of EMA-1 specific- antibodies in the serum samples. The assay was performed in accordance with the manufacturer’s instructions, and the results were reported as % inhibition. Samples that yielded ≥ 40% inhibition were considered positive while samples that exhibited inhibition of < 40% were considered negative.

### 2.6 Statistical analyses

Two-way ANOVA available in GraphPad Prism 10.2.1 (GraphPad Software, San Diego, CA) was used to perform statistical analyses at a significance level (α) of 0.05.

## 3 Results and discussion

Pro-inflammatory cytokines contribute towards reduction in the load of pathogens during infections, however as an undesirable byproduct, they also induce symptoms such as fever, lethargy, anorexia, diarrhea and pain in the host (15). Cytokine storms may also occur resulting in vascular occlusion, vasodilatory shock, hypotension, dyspnea and hypoxemia (17). These symptoms are typically observed during acute *T. equi* infection in addition to severe anemia, peripheral edema, thrombocytopenia, tachycardia, tachypnea and general weakness (31). Severe cases of *T. equi* result in ataxia, pneumonia, cardiac arrhythmias, myalgia, enteritis, laminitis, seizures, and death, in the most extreme cases (31). The objective of this study was to determine whether the manifestation of clinical symptoms that are typically observed during the merozoite stages of *T. equi* infection, occur not only due to hemolysis but also as a result of pro-inflammatory cytokine responses. We also assessed whether anti-inflammatory cytokine expression occurs during the course of infection.

### 3.1 Disease dynamics during primary and secondary T. equi infection

**Figure 2:**
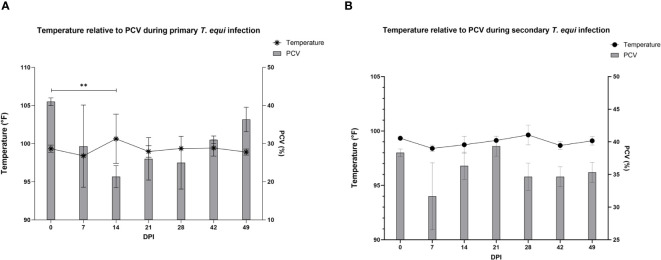
Evaluation of the relationship between PCV and febrility during primary and secondary *T. equi* exposure. **(A)** Decline in PCV was observed during primary *T. equi* infection with a significant decline observed at 14 DPI (**P=0.0098) which coincided with peak febrility. **(B)** No significant difference was observed in PCV levels and temperature during secondary infection.

**Figure 3:**
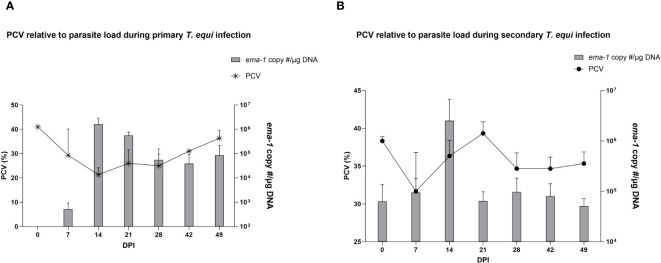
Assessment of the effect of parasite load on PCV during primary and secondary *T. equi* infection. **(A)** An increase in parasite load correlates with a decline in PCV levels in the primarily infected horses indicating the hemolytic effect of merozoites during *T.equi* infection. **(B)** An inverse relationship is observed between PCV and parasite load during secondary *T. equi* infection.

The schizont and pre-schizont stages of *T. equi* development were not evaluated as part of this study. However, the horses manifested typical equine piroplasmosis symptoms and disease dynamics (**Supplementary Table 1**). As expected, a drastic decline in PCV was observed in the primarily infected horses compared to secondarily infected horses. The decline was observed as soon as 2 DPI and by 7 DPI, HO-402’s PCV had decreased by more than 50% (**Supplementary Table 1**). By 16 DPI, all the horses in the primary infected group had a decline in PCV of greater than 50% with a significant decline observed at 14 DPI (P=0.0098) (**Figure 2A** and **Supplementary Table 1**). On the other hand, PCV remained mostly within the normal range of 30% to 47% in the secondary infected horses (**Supplementary Table 1**) and no significant difference was observed in PCV levels pre- and post- secondary infection (**Figure 2B**). Peak rectal temperature coincided with significant decline in PCV in the primary group (P=0.0098) (**Figure 2A**). Similarly, an inverse relationship was observed between PCV and parasite DNA load (**Figure 3**). The highest parasite DNA load assessed by quantification of DNA was observed at 14 DPI in both groups and this coincided with pyrexia in HO-402 (**Supplementary Table 1**).

While the primarily infected animals manifested clinical signs such as anorexia, lethargy, malaise, and pale mucous membranes, none of the secondary infected animals showed these clinical signs. This is possibly because the primary exposure to the *T. equi* merozoite stabilate induced protective immune responses against the parasite (13). In this regard, the parasite DNA load in the secondarily infected group was much lower than in the primarily infected group. This resulted in less severe hemolysis and thus minimal reduction in PCV compared to the primary infected animals (**Supplementary Table 1**). Collectively, symptoms observed in the primary infected group are consistent with the undesirable effects of pro-inflammatory responses. This prompted us to investigate the patterns of expression of cytokines that could have influenced the development of the observed symptoms.

### 3.2 Anti-inflammatory responses are significantly produced during the merozoite stages of T. equi infection in horses

Although the primary infected horses manifested symptoms that might be linked to inflammation, they did not express many of the pro-inflammatory cytokines whose expression was assessed i.e. IL-1β, IFN-γ, IL-6 and TNFα (**Table 1**). Minimal levels of IL-17A, IL-12 and RANTES were expressed by one animal each (**Table 1**), and there was no obvious correlation between their expression and the symptoms observed (**Supplementary Table 1**). Notably, expression of IL-10 was observed in two of the three primary infected horses i.e. HO-402 and HO-396 with significant concentrations observed in the latter (**Table 1**).

**Table 1:** Summary of cytokine expression during primary *T. equi* exposure.

	Animal ID	Days p.i.	Cytokine Concentration (pg/ml)								
			IL-10	IL-1β	IL-6	IL17a	IL-4	IL-12	IFN-γ	TNF-α	RANTES
Primarily Infected Horses		0	-	-	-	-	-	-	-	-	-
		7	-	-	-	-	-	-	-	-	-
		14	-	-	-	-	-	-	-	-	-
	402	21	40.40	-	-	-	-	-	-	-	0.86
		28	31.60	-	-	-	-	-	-	-	-
		42	-	-	-	-	-	-	-	-	-
		49	20.56	-	-	-	-	-	-	-	-
		0	-	-	-	-	-	-	-	-	-
		7	-	-	-	-	-	-	-	-	-
		14	272.92	-	-	-	-	-	-	-	-
	396	21	-	-	-	-	-	-	-	-	-
		28	60.16	-	-	-	-	-	-	-	-
		42	-	-	-	4.95	-	-	-	-	-
		49	-	-	-	4.47	-	-	-	-	-
		0	-	-	-	-	-	-	-	-	-
		7	-	-	-	-	-	-	-	-	-
		14	-	-	-	-	-	-	-	-	-
	398	21	-	-	-	-	-	-	-	-	-
		28	-	-	-	-	-	-	-	-	-
		42	-	-	-	-	-	+	-	-	-
		49	-	-	-	-	-	-	-	-	-

“-” means no cytokine expression was observed.

**Figure 4:**
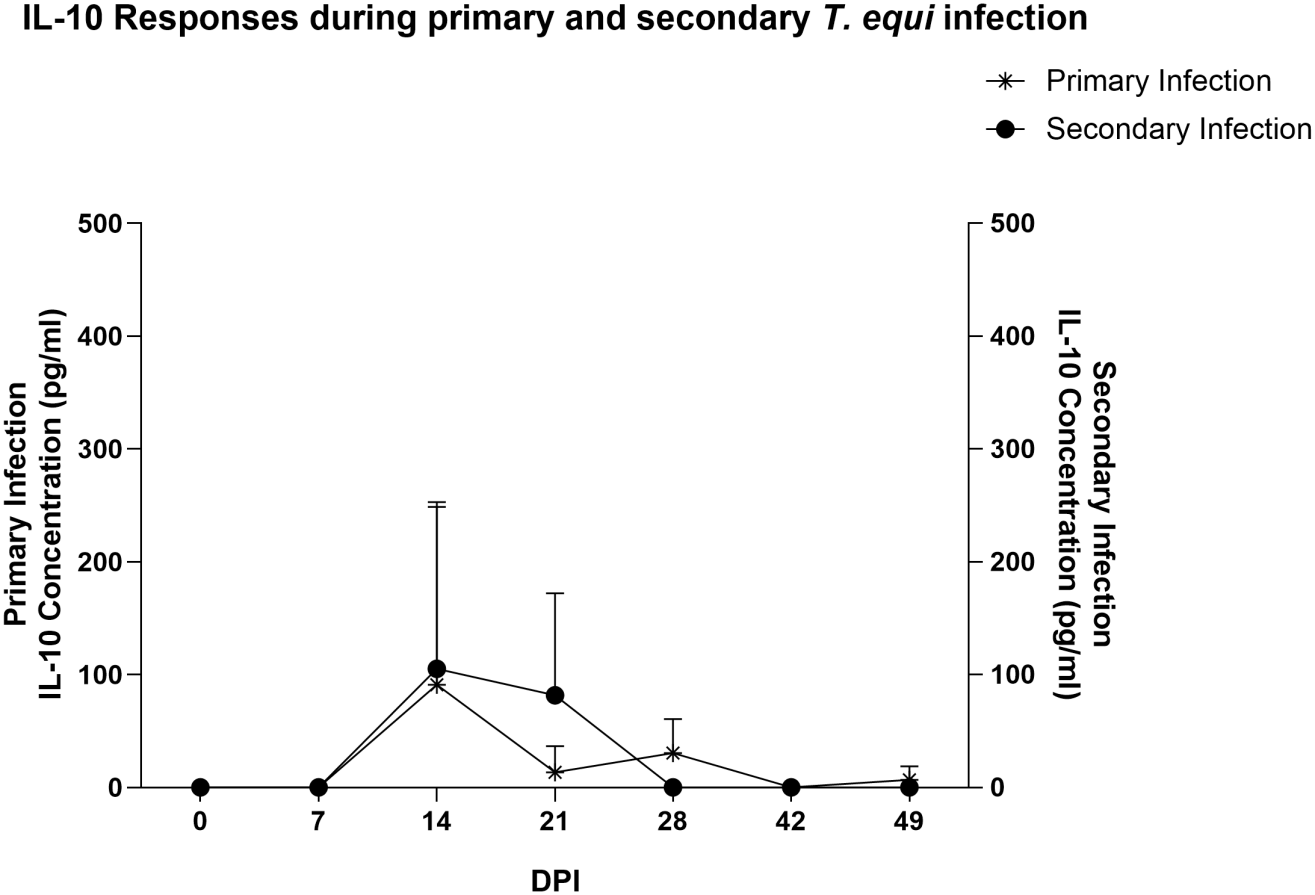
Expression of IL-10 during the merozoite stages of primary and secondary *T. equi* infection. IL-10 was expressed at higher levels in the secondary infected group than in the primarily infected horses. Peak expression was observed at 14 DPI in both cases.

Similarly, most of the pro-inflammatory cytokines i.e., IL-1β, IL-6, IL-17A, IFN-γ, IL-4 and IL-12 were not expressed in the secondarily infected group, except for RANTES and TNF-α. (**Table 2**) The former was expressed at minimal levels by two of the three animals while the latter was expressed by HO-394 only (**Table 2**). There was also no correlation between expression of the pro-inflammatory cytokines and symptoms observed except for elevated temperature and expression of RANTES by HO-394 at 28 dpi (**Supplementary Table 1**). Like the primarily infected group, expression of IL-10 was observed in all the persistently infected horses (**Table 2**). The cytokine was expressed at higher concentrations in the secondarily infected horses than in the primarily infected horses at 14 and 21 DPI (**Figure 4**). Additionally, the highest concentrations of the cytokine were observed at 14 DPI, and this coincided with the maximum parasite load in both groups (**Figure 3**).

**Table 2:** Expressed cytokines during secondary *T. equi* infection.

	Animal ID	Days p.i.	Cytokine Concentration (pg/ml)								
			IL-10	IL-1β	IL-6	IL17a	IL-4	IL-12	IFN-γ	TNF-α	RANTES
Secondary Infected Horses		0	-	-	-	-	-	-	-	-	-
		7	-	-	-	-	-	-	-	-	-
		14	-	-	-	-	-	-	-	-	0.78
	397	21	185.79	-	-	-	-	-	-	-	-
		28	-	-	-	-	-	-	-	-	-
		42	-	-	-	-	-	-	-	-	-
		49	-	-	-	-	-	-	-	-	-
		0	-	-	-	-	-	-	-	-	-
		7	-	-	-	-	-	-	-	-	-
		14	274.05	-	-	-	-	-	-	-	-
	403	21	22.76	-	-	-	-	-	-	-	-
		28	-	-	-	-	-	-	-	-	-
		42	-	-	-	-	-	-	-	-	-
		49	-	-	-	-	-	-	-	-	-
			-	-	-	-	-	-	-	-	-
		0	-	-	-	-	-	-	-	-	-
		7	-	-	-	-	-	-	-	-	1.12
		14	41.90	-	-	-	-	-	-	3.5	-
	394	21	37.04	-	-	-	-	-	-	-	-
		28	-	-	-	-	-	-	-	-	1.33
		42	-	-	-	-	-	-	-	51.4	0.40
		49	-	-	-	-	-	-	-	13.13	-

“-” means no cytokine expression was observed.

**Figure 5:**
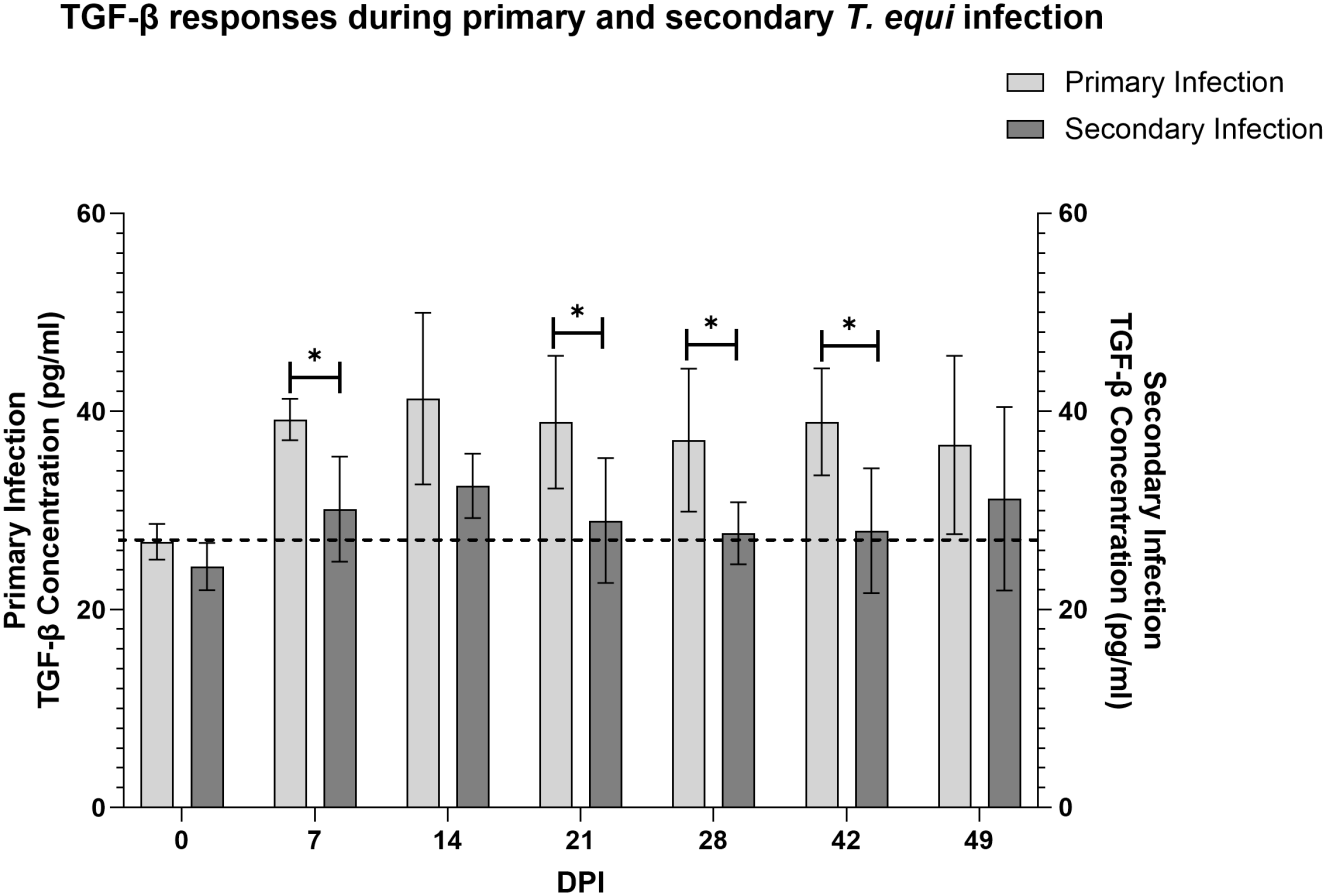
Evaluation of expression of TGF-β1 during primary and secondary *T. equi* infection. TGF-β1 was expressed at higher levels during primary *T. equi* infection than secondary infection. * p<0.0456. The cut-off value is represented by the dotted line at 26.8 pg/ml.

Analysis of expression of TGF-β1 using a competitive inhibition ELISA showed that all the horses in this study expressed the cytokine. Primarily infected animals expressed higher levels of the cytokine compared to secondarily infected horses especially at 7, 21, 28 and 42 DPI (**Figure 5**). Notably, significant expression of TGF-β1 was observed at 7 and 14 DPI in the primarily and secondarily infected groups respectively (* p<0.0213). Like IL-10, the highest levels of TGF-β1 expression in both groups were observed at 14 DPI which coincided with the highest parasite load (**Figure 3**).

IL-10 is a potent anti-inflammatory cytokine that limits the detrimental effects of host pro-inflammatory responses to pathogens thus averting tissue damage (32). The cytokine was originally considered to be primarily produced by CD4 T_H_2 and CD4 T regulatory (T_reg_) cell subset but recent studies have shown that it can also be produced by many categories of leukocytes, with the major sources being T helper cells, monocytes, macrophages and dendritic cells (32). Studies have also indicated that IL-10 is a key molecule in underpinning B cell differentiation, proliferation and antibody production thus promoting humoral immune responses (33).

TGF-β regulates several T cell functions including inhibition of the differentiation of T helper 1 (Th1) and T helper 2 (Th2) cells and promotion of development of T_reg_ and T follicular helper (T_fh_) cells (34). These are essential in promoting anti-inflammatory responses (35), and antibody responses (36), respectively.

**Figure 6:**
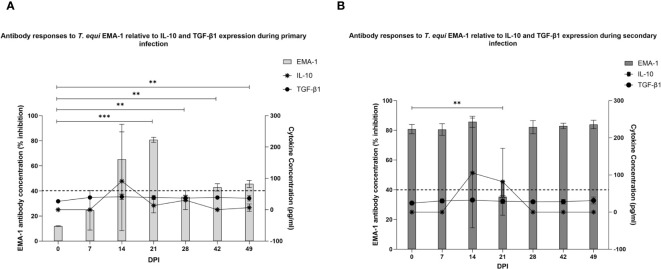
Assessment of the relationship between IL-10 and TGF-β expression and EMA-1 antibody production. **(A)** EMA-1 antibody production was first observed at 14 DPI during primary *T. equi* infection. Significant expression levels were observed at 21 to 49 DPI. ** p<0.0079, *** p=0.0006. The increase in antibody production at 14 DPI coincided with increased expression of IL-10 and TGF-β1. **(B)** EMA-1 antibodies were present in the secondary-infected group at 0 DPI due to *T. equi* pre-exposure. A slight peak in EMA-1 antibody levels was observed at 14 DPI coinciding with high levels of IL-10 and TGF-β expression. A significant decline in antibody levels was observed at 21 DPI. ** p=0.0047. In both cases, the dotted line represents the antibody ELISA cut-off value of 40% inhibition.

Since pro-inflammatory cytokines were not consistently expressed in this study, and very low levels were observed in cases where expression occurred, it is likely that the high levels of IL10 and TGF-β1 suppressed the pro-inflammatory cytokines to below detection levels. There is also the probability that expression of the anti-inflammatory cytokines was not associated with modulation of pro-inflammatory responses. In this regard, we investigated the possibility of an association between expression of the anti-inflammatory cytokines and development of antibodies by evaluating the production of antibodies against *T. equi* EMA-1 protein. Our findings showed significant production of antibodies against *T. equi* during primary infection at 21 to 49 DPI (**Figure 6A**). As expected, antibodies against EMA-1 were present in the secondary infected group at 0 DPI and no significant increase in antibody production was observed in this group except for a significant decline at 21 DPI (**Figure 6B**). Existence of a correlation between IL-10 and TGF-β expression, and EMA-1 antibody production was observed, with increasing antibody levels coinciding with high expression of the cytokines (**Figures 6A, B**) and parasite DNA levels at 14 DPI. This is consistent with our previous findings which showed that significant antibody responses against the rhoptry-associated proteins were observed in these animals, and high levels were also observed at 14 DPI (13). The direct relationship between high parasite load and expression of IL-10 and TGF-β further suggests that the cytokines were produced in response to the high level of parasites thus leading to antibody production and subsequent reduction in the parasite load.

Collectively, unlike the findings observed in studies involving transforming *Theileria* species (23, 37), significant pro-inflammatory responses were not observed in this study. Additionally, our findings were contrary to the observations made on serological levels of cytokines during natural *T. equi* infection (27). Notably, this could be due to the fact that this study focused solely on the merozoite stages of *T. equi* development thus suggesting that the cytokine responses observed by Mostafavi et al. could be associated with schizogony and thus further studies are required to ascertain if this is the case.

The findings obtained from this study provide insight into the relationship between *T. equi* merozoites and its hosts by highlighting the cell mediated immune responses that occur in response to the parasite. The study demonstrates that merozoites trigger substantial anti-inflammatory responses and minimal pro-inflammatory responses, strongly suggesting that the symptoms observed during primary exposure to the parasite can be associated with hemolysis and subsequent anemia, but not pro-inflammatory responses. It is worth noting that even though hematocrit was stable in secondarily infected horses, the slight drop observed in one of the horses suggests that some carrier animals that are clinically inapparent might not exhibit a very strong immune response thus posing challenges to the equine industry. The host cellular immune response to parasites that transiently infect nucleated equid host cells such as *T. equi* and related *Theileria*-like apicomplexan parasites is technically difficult to investigate. However, from the current study it seems clear that expression of IL-10 and TGF-β in the merozoite stages of infection are very likely associated with stimulation of antibody production, but not dampening of pro-inflammatory immune responses even though both pro- and anti- inflammatory cytokines were co-expressed in some cases. Notably, it is likely that the changes in cytokine levels could have occurred due to cell activation. Further studies are therefore required to confirm if this could be a factor in this study. Additional studies are also required to evaluate recall of cytokine responses by re-stimulating immune cells from *T. equi*-infected horses *in vitro*.

## Data availability statement

The original contributions presented in the study are included in the article/**Supplementary Material**. Further inquiries can be directed to the corresponding author.

## Ethics statement

The animal study was approved by the Institutional Animal Care and Use Protocol Committee of the University of Idaho (protocol #2018-19). The study was conducted in accordance with the local legislation and institutional requirements.

## Author contributions

CO: Conceptualization, Data curation, Formal analysis, Investigation, Methodology, Supervision, Validation, Visualization, Writing – original draft, Writing – review & editing. RGB: Writing – review & editing. RPB: Writing – review & editing. CS: Conceptualization, Formal analysis, Funding acquisition, Investigation, Methodology, Project administration, Resources, Supervision, Validation, Visualization, Writing – review & editing. LF: Conceptualization, Formal analysis, Funding acquisition, Investigation, Methodology, Project administration, Resources, Supervision, Validation, Visualization, Writing – review & editing.
